# A tailored nanocarrier DMON and CpGs synergistically drive the formulation of a highly immunogenic and long-acting vaccine against echinococcosis

**DOI:** 10.1016/j.mtbio.2025.101868

**Published:** 2025-05-12

**Authors:** Ting Xin, Xintao Gao, Siyi Tao, Chenghao Zhou, Zhifang Zhang, Jiabo Ding, Jiaxi Ru, Yinü Li

**Affiliations:** a Institute of Animal Science, Chinese Academy of Agricultural Sciences, Beijing, 100193, China; b National Key Laboratory of Agricultural Microbiology, Biotechnology Research Institute, Chinese Academy of Agricultural Sciences, Beijing, 100081, China; c Institute for Advanced Research, Cixi Biomedical Research Institute, Wenzhou Medical University, Zhejiang, 325035, China

**Keywords:** Nanocarrier, DMON nanoparticle, CpG adjuvants, Vaccine, Echinococcosis, Early, robust and long-lasting immunity

## Abstract

Hydatid disease (echinococcosis) is a zoonotic parasitic disease that seriously endangers human health and livestock production. To develop a safer, more effective vaccine with an exceptionally long-lasting immune response, we employed an ‘all-in-one’ strategy to construct a nanovaccine against echinococcosis. In this system, a dendritic mesoporous organosilica nanoparticle (DMON), Eg95 antigen, and two types of CpG potentiators (CpG ODN and pCpG) were integrated into a single nanoplatform. Compared to the commercial Quil-A-formulated vaccine, these two nanovaccines exhibited significant advantages in inducing early, robust, and long-lasting protective immune responses, especially in terms of IgG1 antibody responses and Th1 cytokine TNF-α secretion. Notably, the certain adjuvant combination (DMON + pCpG) formulated-vaccine Eg95N + pCpG@DMON conferred stronger adjuvanticity to the antigen than Quil-A during the late stage (42–84 days). Systematic evaluation demonstrated excellent biodegradability and biosafety of DMON and its-based vaccine. This research provides a strong foundation for upgrading vaccines against echinococcosis.

## Graphical abstract

**Figure:**
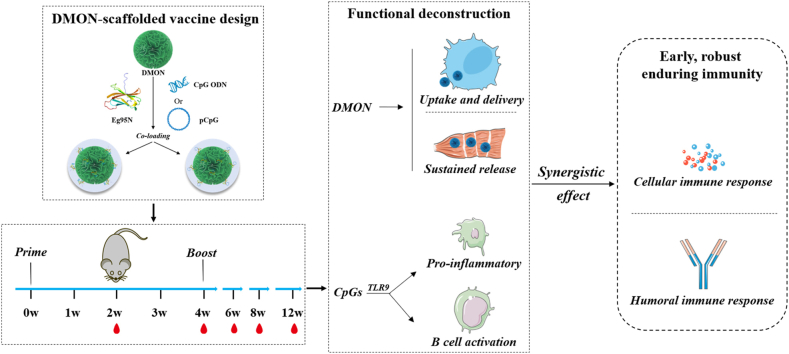

## 1 Introduction

Hydatid disease (Echinococcosis) is a global zoonotic parasitic disease primarily caused by infection with the larvae of the cestode *Echinococcus granulosus (E*. *granulosus)*, and has been listed as one of the 20 neglected tropical diseases [1,2]. Echinococcosis is categorized into cystic echinococcosis (CE), alveolar echinococcosis (AE), and polycystic echinococcosis. Among them, CE is cosmopolitan, posing a significant threat to human health and the livestock industry [[3], [4], [5], [6]]. Vaccination is the most cost-effective strategy for preventing CE infection [7]. Currently, recombinant hydatids subunit vaccine contain Eg95 antigen and Quil-A adjuvant have demonstrated over 90 % protective efficacy in livestock, specifically sheep and cattle [[8], [9], [10]]. Although Quil-A exhibits robust immunostimulatory effect, it may also trigger side effects, such as hemolysis and tissue necrosis, thereby impeding the vaccine's use in humans [11]. Therefore, the development of a safe and highly effective vaccine adjuvant is highly desirable for combating hydatid disease.

Adjuvants can boost antigen-specific immune responses, thereby promoting vaccine efficacy and dose-sparing capability while reducing costs and potential side effects [12]. A dual-functional adjuvant system integrating delivery and immune potentiation conforms to the advancement requirements of vaccinology. CpG oligonucleotide (ODN), a kind of Toll-like receptor 9 (TLR9) agonist, proves to be a safe and promising adjuvant for the development of new-generation vaccines and has been used in an approved recombinant hepatitis B vaccine (HEPLISAV-B®) [13]. DMON, a rising mesoporous biomaterial, possess three-dimensional (3D) center-radial nanochannels and organic components within its framework. These features endow them with unique pore structures (e.g., larger pore volumes, more open pore channels, and more accessible internal spaces), enhance their biocompatibility, hydrophobicity, and biodegradability compared to conventional mesoporous silica nanoparticles (MSNs) [14], and enable the application in loading anti-cancer or -pest drugs [15], showing good agent-delivery efficacy [16,17]. To the best of our knowledge, however, the application of DMONs in vaccines for Echinococcosis has not been reported until now.

Since the discovery of their immunostimulatory activity in 1995 [18]. CpG ODNs have achieved certain success as a vaccine adjuvant, with over 1500 preclinical research articles published on CpG ODNs-based vaccines against a plethora of pathogens. Nevertheless, their synthesis and purification processes impose cost barrier for popularization and application in veterinary field. pCpG is a plasmid DNA containing 20 copies of CpG motifs that offers robust immunostimulatory activity [19,20] with significant cost advantage and ease of production on a large scale. While CpG ODN (or pCpG) effectively enhance both antigen-specific humoral and cellular responses, their adjuvanticity to the Eg95 antigen remains inferior to that of Quil-A [21]. This limitation highlights the critical need to optimize the efficacy of the vaccine employing the CpG ODN/pCpG + Eg95 formulation.

The combination adjuvant, composed of delivery system and potentiator(s), giving rise in synergistic immune activation and the amplified immune response [22]. In light of the advantages of DMON as a nanocarrier, as well as the insufficient adjuvanticity of CpGs to Eg95 antigen, tailored DMON and CpG ODN (or its derivative, pCpG) were employed together in this study. We hypothesize that the leverage of the ultra-large pore structure of DMON enables the co-loading of Eg95 antigen and potentiator(s) within a single nanoparticle, thus providing a viable avenue for the development of a cost-effective vaccine against echinococcosis.

## 2 Experimental section

### 2.1 Materials

Quil-A adjuvant was purchased from InvivoGen (San Diego, CA). The CpG-enriched pUC18 plasmid containing 20 copies of CpG ODN 2006 (sequence: 5′-TCGTCGTTTTGTCGTTTTGTCGTTTTGTCGTTTTGTCGTT-3′) which belongs to class B CpG, was constructed, purified and stored at our laboratory [19,20]. A class B CpG ODN 1826 (GACGTT) [23] containing two CpG motifs embedded in a phosphorothioate backbone, was synthesized by Sangon Biotech (Beijing, China). BHK-21 cells (CCL-10), MDBK cells (CCL-22), and RAW 264.7 cells (TIB-71) were obtained from American Type Culture Collection (ATCC). BALB/c female mice (6–8 weeks old) were obtained from Vital River Laboratory Animal Technology Co., Ltd. (Beijing, China).

The expression and purification of soluble Eg95N antigen was performed as follows. The truncated Eg95 (14-129aa) with a GST tag at the N-terminus (Eg95N-GST) was expressed in *Escherichia coli* (*E. coli*) BL21 (DE3) and purified using a Glutathione Sepharose 4B affinity chromatography column (Cytiva, Germany); after digestion with HRV 3C protease (Thermofisher, USA) and purification by ion-exchange chromatography (HiTrap Capto SP ImpRes Column, Cytiva, Germany) and size exclusion chromatography (Superdex 75 10/300 GL column, Cytiva, Germany), GST-free Eg95N (Eg95N) was finally obtained from Eg95N-GST. The endotoxin removal from protein was performed twice and the removal effect was confirmed, as described previously [24]. The protein Eg95N was filter-sterilized, quantified by BCA assay, and then stored at −80 °C for further use.

### 2.2 The synthesis and characterization of DMONs

Firstly, 950 mg of cetyltrimethylammonium bromide (CTAB, Macklin, China) and 500 mg of sodium salicylate (NaSal, Macklin, China) were added to 60 mL of water under gentle stirring in an oil bath at 80 °C until fully dissolution (approximately 2 h). Next, 150 mg of triethanolamine (TEA, Macklin, China) was added to the solution with continuous stirring for 30 min. Subsequently, 5 mL of tetraethyl orthosilicate (TEOS, Macklin, China) and 4 mL of 1,2-bis(triethoxysilyl)-ethane (BTEE, Macklin, China) were introduced into the reaction system. The mixture was maintained 80 °C with stirring (700 rpm) for 12 h. The resultant nanoparticles were collected through high-speed centrifugation (10000 rpm, 10 min), followed by three cycles of ethanol hydrochloric acid treatment to remove residual reactants.

### 2.3 Protein and CpGs loading and release

DMONs were dispersed in 10 mM phosphate-buffered saline (PBS, pH 7.4) to prepare a nanoparticle suspension (1 mg/mL). subsequently, 0.5 mL of the prepared suspension was mixed with an equal volume of Eg95N (1 mg/mL) under gentle agitation (200 rpm) at 4 °C for 14 h. After encapsulation, the soluble Eg95N in the supernatant was isolated by centrifugation and its concentration was quantified using the Pierce™ BCA Assay Kit (Thermo Fisher Scientific, USA). The Eg95N loading ratio was calculated by the following formula:

$$\text{loading}\;\text{ratio}\;\left(\frac{\mu g}{\text{mg}}\right)=\frac{\text{initial}\;\text{protein}\;\text{amount}\;\left(\mu g\right)–\;\text{protein}\;\text{amount}\;\text{in}\;\text{the}\;\text{supernatant}\;\left(\mu g\right)}{\text{amount}\;\text{of}\;\text{DMONs}\;\left(\text{mg}\right)}\text{.}$$

For the optimal loading ratio, Eg95N@DMON complexes with mass ratios ranging from 1:1 to1:10 (antigen: carrier) were prepared by mixing 200 μL of Eg95N solution (250 μg/mL) with an equal volume of DMONs at serially diluted concentrations (250–2500 μg/mL). After encapsulation and isolation as described above, both loaded Eg95N in the precipitate and soluble Eg95N in the supernatant were analyzed by 12 % SDS-PAGE. Concurrently, soluble protein was also quantified using the Pierce™ BCA Assay Kit. To characterize the release profile of Eg95N *in vitro*, the loaded Eg95N in the precipitate obtained above was dispersed in 0.4 mL PBS and incubated at 4 °C with shaking. At predetermined time intervals, the suspension was centrifuged at 12,000 rpm for 10 min, to isolate and remove the supernatant (400 μL), which was replaced with an equal volume of fresh PBS. The concentration of Eg95N in the supernatant were measured using the Pierce™ Micro BCA Protein Assay Kit.

The loading and release characteristics of CpG ODN and pCpG were also examined. DMONs (100 μL) with varying concentrations (1, 2, 3, 4, 5, 6, 7, 8, 9, 10 mg/mL) was mixed with 100 μL of CpGs (100 μg/mL). After mixing evenly, the mixtures were incubated at 4 °C for 14 h, the soluble CpG ODN and pCpG in the isolated supernatants were detected by Nanodrop. DMONs (1 mg/mL, 200 μL) was mixed with 200 μL of CpGs (100 μg/mL) and the mixture was incubated at 4 °C for 14 h. After centrifugation (1000 g, 10 min), the supernatant (0 h) was transferred to a new tube and performed with Nanodrop determination. The precipitate was resuspended with 200 μL PBS at 4 °C and the soluble CpGs in supernatants at 2 h, 4 h, 6 h, 8 h, 10 h, 24 h, 48 h were quantified using Nanodrop.

### *2.4* *In vitro**cellular*** uptake and *in vivo* trafficking

To investigate the *in vitro* cellular uptake and *in vivo* trafficking of Eg95N and CpGs, DMON, pCpG and Eg95N were labeled with Cy3 (Invitrogen, USA), Cy5 (Label IT® Cy5 Nucleic Acid Labeling Kit, Mirus Bio, USA) and fluorescein isothiocyanate (FITC, Invitrogen, USA), respectively, according to the manufacturer's instructions. 5′-Cy5-labeled phosphorothioated CpG ODN was synthesized by Sangon Biotech (Being, China). After fluorescent labeling, Eg95N-FITC and pCpG-/CpG ODN-Cy5 were co-loaded onto DMON-Cy3. RAW 264.7 cells were seeded onto 8-chamber confocal coverglass at 2 × 10^5^ cells/mL (400 μL/well) overnight. After washing, cells were incubated with Eg95N-FITC (5 μg/mL), CpGs-Cy5 (3 μg/mL), DMON-Cy3 (50 μg/mL), or Eg95N-FITC + CpGs-Cy5@DMON-Cy3 at 37 °C for 3 h. The images were captured using an LSM 710 confocal laser scanning microscope (Zeiss, Germany).

BALB/c mice (n = 3) were immunized intramuscularly in thigh with 0.1 mL of Eg95N-FITC (30 μg), Cy5-labeled CpGs (10 μg), or Eg95N-FITC + CpGs-Cy5@DMON, and then imaged at 0.5 h (2 h), 1 h (24 h), 2 h (48 h) and 6 h (96 h) using an AniView 100 *in vivo* imaging system (IVIS) (BLT Photon Technology, China) after anesthesia with isoflurane.

### 2.5 Vaccine formulation and animal immunization

The formulations of the different vaccine groups were shown in Table 1. Soluble Eg95N and/or CpG adjuvant were loaded onto DMONs by incubating the mixture of all components at 4 °C, with gentle agitation (200 rpm) for 14 h, to form nanovaccines (G5-G7). Eg95N + Quil-A (G2) was used as the positive control group, while PBS served as the negative control. Six-week-old female BALB/c mice were randomly divided into eight groups (n = 6), and immunized intramuscularly in thigh two time at a four-week interval with 0.1 mL. All animal experiments were performed in accordance with the guidelines of the Institutional Animal Care and Use Committee of Institute of Animal Science (approval number: IAS2024-108), and approved by the Animal Ethical Welfare Committee of IAS.

**Table 1:** Vaccine formulation and experimental grouping.

Groups	Contents	Eg95N (μg/mouse)	Quil-A (μg/mouse)	CpG ODN (μg/mouse)	pCpG (μg/mouse)	DMONs (μg/mouse)
G1	Eg95N	15	/	/	/	/
G2	Eg95N + Quil-A	15	15	/	/	/
G3	Eg95N + CpG ODN	15	/	5	/	/
G4	Eg95N + pCpG	15	/	/	10	/
G5	Eg95N@DMONs	15	/	/	/	150
G6	Eg95N + CpG ODN@DMONs	15	/	5	/	150
G7	Eg95N + pCpG@DMONs	15	/	/	10	150

### 2.6 Humoral immune responses of the DMON-scaffolded vaccine

Serum samples were harvested from the immunized mice at 14, 28, 42, 56, 84 days post-primary vaccination (dpv) to measure the Eg95-specific antibodies via indirect ELISA. Briefly, Eg95N dissolved in 0.05 M carbonate buffer (pH 9.6) was added into the 96-well ELISA plate (0.1 μg/well) and incubated at 4 °C overnight. The plate was blocked with PBS containing 5 % milk (0.2 mL/well) at 37 °C for 2 h. After incubation with serially diluted serum for 1 h at room temperature (RT), the plate was sequentially washed six times with PBS containing 0.05 % Tween-20 (PBST, 0.2 mL/well), incubated (1 h, RT) with HRP-conjugated goat anti-mouse IgG, IgG1, IgG2a, IgG2b, or IgG3 (0.1 mL/well), washed six times with PBST (0.2 mL/well), and incubated (15 min, RT) with TMB substrate solution (0.1 mL/well). The reaction was stopped with 1 M HCl (50 μL/well), and the optical density (OD) at 450 nm was measured.

### 2.7 Cellular immune responses of the DMON-scaffolded vaccine

#### 2.7.1 Granzyme B and cytokine secretion assays

All mice were euthanized at 84 dpv, and spleens were collected and minced to harvest the splenocyte. After three washes with Dulbecco's Modified Eagle Medium (DMEM) containing antibiotics-antimycotics, the splenocytes were performed with RBC removal according to the introduction of RBC lysis, and then the viable cell resuspended in 1 mL DMEM were counted using 0.2 % Trypan blue (Gibco-BRL) with a Neubauer counting chamber. The 2 × 10^4^ cells/well in DMEM (10 % FBS, 10 mM HEPES, 1 % antibiotics) were and seeded in 96-well plate. After adhesion, cells were stimulated for 72 h at 37 °C under 5 % CO_2_ with Eg95N (10 μg/mL), BSA (10 μg/mL), ConA (5 μg/mL) or PBS respectively. The levels of Granzyme B, IFN-γ, IL-4, IL-6, IL-10 and TNF-α in the supernatants were analyzed with MILLIPLEX™ MAP mouse CD8^+^ T cell Magnetic bead panel (Merck, Germany) according to the manufacture's introduction.

#### 2.7.2 The proliferation of splenetic lymphocytes

The seeded splenocytes (2 × 10^5^ cells/mL) in 96-well plates (0.1 mL/well) were stimulated with Eg95N, BSA, ConA, or blank control for 72 h at 37 °C, 5 % CO_2_. Subsequently, MTS stock solution (10 μL/well) was added to each well, followed by incubation at 37 °C under 5 % CO_2._ After incubation for 3 h, optical density (OD) at 490 nm was measured and the stimulation index (SI) was calculated by the formula: $\text{SI}=\frac{ConA\;or\;BSA\;or\;Eg95-Blank}{Control-Blank}\times 100\%$.

### 2.8 Safety evaluation

#### 2.8.1 Hemolysis assay of DMONs

Fresh blood samples were collected from guinea pig heart to prepare the red blood cells (RBCs), which was washed five times and then diluted with PBS (1:10) for further use. The diluted RBC solution (0.3 mL) was added into 1.2 mL of PBS solution containing serially diluted DMONs (50, 100, 200, 400, 800, and 1000 μg/mL), or into equal volumes of deionized water and PBS as positive and negative controls, respectively. The mixtures were gently shaken and then quiesced at RT for 2 h. Finally, the supernatant sample from the mixture was isolated by centrifugation and its absorbance at 541 nm was measured using a Shimadzu UV-2500 UV–vis spectrophotometer. The hemolysis rate (HR) of DMONs was calculated as follows: $\text{HR}=\frac{A\;sample-A\;negative\;control}{A{\;}_{positive\;control}-A{\;}_{negative\;control}\;}\times 100\%$.

#### 2.8.2 MTS analysis

RAW 264.7, MDBK and BHK-21 cells were seeded into 96-well culture plates (1 × 10^4^ cells/well) respectively. After cell attachment, DMONs with gradient concentration (12.5, 25, 50, 100, 200, 400 μg/mL) were added into cells and incubated at 37 °C under 5 % CO_2_. After incubation for 24 h, the cells were treated with 10 μL of MTS stock solution for another 3 h, and then optical density (OD) at 490 nm was measured.

#### 2.8.3 Histopathology

At 84 dpv, mice were sacrificed, dissected, and tissue samples including heart, liver, spleen, lung, kidney and the injection site of muscles were collected. Sequentially, samples were fixed, embedded, sectioned, and stained to obtain the histological sections, which were then observed by a light microscope.

#### 2.8.4 Serum biochemical test

The blood biochemical indicators including alanine aminotransferase (ALT), aspartate aminotransferase (AST), urine albumin excretion rate (UREA) and creatinine (CREA) in serum samples harvested at 84 dpv, were detected by Chemray-240 automatic clinical blood chemistry analyzer (Rayto, China).

### 2.9 Statistical analysis

The statistical significance of the data was evaluated by one-way ANOVA or unpaired two -tailed *t*-test using GraphPad Prism 9.0. A *p*-value <0.05 was considered statistically significant.

## 3 Results and discussion

### 3.1 Design of echinococcosis nanovaccines

The life cycle of *E*. *granulosus* is quite complex, involving six forms of the parasite and requires the participation of both intermediate and definitive hosts [6]. Sheep serving as the main intermediate, are globally farmed and can get sick after infection. However, current vaccine for sheep use Quil-A as an adjuvant, which has serious side effects (e.g., hemolysis and tissue necrosis), and high production costs; moreover, geographical inconvenience and economic burden posed a demand for reducing the frequency of immunizations. Therefore, new vaccine adjuvants are urgently needed to develop a safer, low-cost subunit vaccine against echinococcosis that can induce long-term protective immunity.

To achieve the aforementioned objectives, we envision employing an ‘all-in-one’ strategy to develop a novel nanovaccine for CE control, wherein the nanocarrier, antigen, and potentiator are co-assembled into a single nanoplatform. In this system, DMONs are selected as the nanocarrier due to their central radial pore structure, which facilitates the efficient loading of bioactive macromolecules. Additionally, their framework contains organic components, making them more hydrophobic, easier to degrade, and offering enhanced biosafety. Therefore, they not only exhibit superior adsorption capability for bioactive macromolecules but also demonstrate high safety.

In 2000, with the discovery of Toll-like receptor 9 (TLR9) that specifically recognizes bacterial DNA [25], the mechanism underlying the adjuvant effect of CpG-ODNs was gradually unveiled. CpG ODNs are internalized and activate TLR9. The activation of TLR9 triggers NF-κB- and interferon regulatory factor (IRF)-mediated pro-inflammatory responses, as well as induces B cell-mediated responses. CpG ODNs, can be broadly categorized into class A (D-type), class B (K-type), class C, and class P. Among them, class B CpG ODN, is the first discovered and most well-studied, and have been widely used in vaccine adjuvant research and approved for use in one human vaccine. To achieve a highly effective and long-lasting protective immune response, a class B CpG ODN was selected as potentiator to enhance the efficacy of the vaccine. However, CpG ODNs are typically synthesized using solid-phase synthesis techniques and require HPLC purification, which results in higher production costs and limits their widespread use in veterinary vaccines. Sato et al. found that the immune efficacy of DNA vaccines is also closely related to the CpG motif on plasmid DNA [26]. Inspired by this, a pCpG was prepared through genetic engineering methods by our team, which not only enables large-scale, low-cost production but also, due to its multiple CpG motifs, exhibits enhanced immunostimulatory effects [19,20]. In this study, considering the cost, this inexpensive pCpG was also employed to compare the adjuvanticity with the normal CpG ODN.

The Eg95 protein is present in the oncosphere, protoscolex and adult intestinal stages [27]. As Eg95 appears not to be expressed on the tegument of the activated oncosphere stage, it is proposed that this infective stage is killed by complement-mediated antibody attack in Eg95-vaccinated sheep during early post-oncospheral development in lung or liver tissues [28]. Although the biological function of Eg95 is mainly unknown, the association between its induced antibody response and protective immunity has been reported [29]. About Eg95-specific immunity, previous studies also revealed that the sheep hydatid fluid elicits both Th1 and Th2 cell activation in human CE. The induction of a cytokine-related Th2 response leads to susceptibility to echinococcosis, but a Th1 response results in protective immunity against the disease. Another study found that Th1 immune response is more efficient than the Th2 one in controlling parasitic infection [30]. Guided by this Th1 preference, a Th1-biased potentiator-class B CpG ODN and its cost-effective derivative were chosen in this study, which also echoes the selection of CpGs type described in the previous paragraph. The current vaccine-used Eg95 antigen is produced as a GST-tagged recombinant fusion protein expressed in inclusion bodies. Standard solubilization-refolding purification protocols, characterized by complexity, time inefficiency, and suboptimal cost-effectiveness [31], yielded target antigen with <25 % purity, leading to substantial batch-to-batch variability [28]. Therefore, a truncated Eg95N in soluble form was designed and employed for all in one vaccine development.

**Fig. 1:**
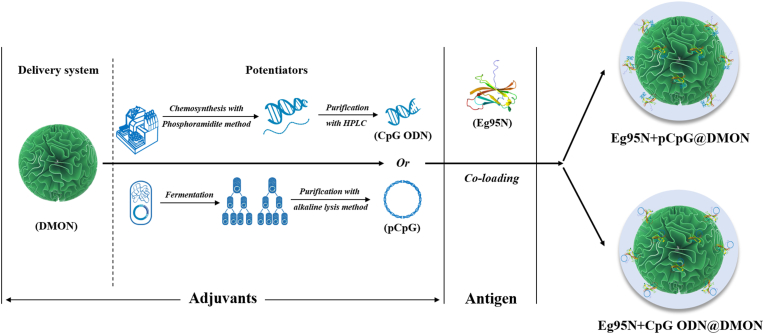
DMON-scaffolded vaccine design.

Taken together, a CpG ODN or its derivative pCpG was employed to co-load with a truncated soluble Eg95N onto DMON to design an ‘all-in-one’ DMON-scaffolded vaccine (Fig. 1).

### 3.2 Preparation and characterization of nanocarrier, antigen, and CpG adjuvants

**Fig. 2:**
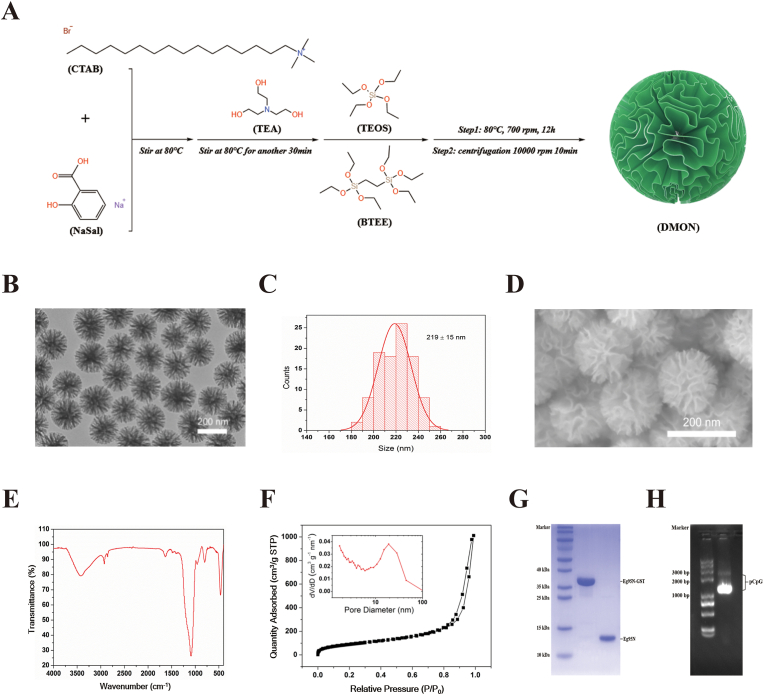
Preparation and characterization of components for vaccine formulation. (A) Synthetic routes of the DMONs. (B) TEM image of DMONs. Scale bars, 200 nm. (C) Size distribution of DMONs measured by TEM. (D) SEM image of the DMONs. Scale bars, 200 nm. (E) FTIR spectra of DMONs. (F) N2 adsorption-desorption isotherm and corresponding pore size distribution curve (inset) of the DMONs. (G) SDS-PAGE analysis of antigen. (H) Agarose gel electrophoresis of pCpG.

Firstly, DMONs were prepared by co-condensing inorganic and organosilica precursors in an aqueous solution, following our previously reported method with minor modifications. In this synthesis system, TEOS and BTEE were used as precursors, CTAB and NaSal served as the template agents, and TEA acted as the alkaline catalyst (Fig. 2A). As depicted in Fig. 2B, the central-radial structure of the synthesized DMONs can be clearly observed through transmission electron microscopy (TEM). The TEM-measured diameter of the synthesized DMONs was 219 ± 15 nm (Fig. 2C). Scanning electron microscopy (SEM) further revealed their spherical morphology, characterized by a radial wrinkle structure and center-radial fibrous pore channels (Fig. 2D). Both TEM and SEM confirmed the excellent monodispersity and uniform size of the synthesized DMONs. To further analyze the organic composition of the DMONs, Fourier-transform infrared spectroscopy (FT-IR) was employed to confirm the presence of -CH_2_-CH_2_- groups. As shown in Fig. 2E, except the characteristic absorption peak of the Si-O-Si bond (1089.70 cm^−1^, 801.11 cm^−1^), a distinct absorption peak at 2924.84 cm^−1^ corresponding to -CH_2_-CH_2_- groups was observed, indicating the presence of ethyl groups within the DMONs framework. To further characterize their pore structures, the N_2_ adsorption-desorption isotherm was analyzed. The isotherm exhibited typical Type IV curves, with a steep capillary condensation step occurring at relative pressures (*P*/*P*_0_) between 0.8 and 0.9. The Brunauer-Emmett-Teller (BET) surface area and total pore volume of the DMONs were determined to be 333 m^2^ g^−1^ and 0.64 cm^3^ g^−1^, respectively, with an average pore size of ∼16 nm (Fig. 2F). These high BET surface area, substantial total pore volume, and highly accessible surface area are very favorable for the loading of large biomolecules. Furthermore, with the introduction of ethyl groups into the DMON framework, the hydrophobicity and biodegradability of the DMONs could be improved (see below).

The truncated Eg95 antigen with a GST tag (Eg95N-GST) was expressed in soluble form using *E. coli* BL21. Following affinity chromatography purification, the GST tag was cleaved by HRV 3C protease to harvest the soluble Eg95N. A single band at about 13 kDa corresponding to Eg95N in SDS-PAGE analysis, showing high purity (Fig. 2G). The fermentation-derived pCpG stored in our laboratory was also verified by agarose gel electrophoresis before use (Fig. 2H), while highly purified phosphorothioated CpG ODN was synthesized by Sangon Biotech (Being).

Taken together, good uniformity and dispersion of synthesized DMONs with a wrinkled radial structure and center-radial pore, as well as high-quality Eg95 antigen and CpG potentiators, lay a good foundation for the successful preparation of the ‘all in one’ vaccine we conceived.

### 3.3 Protein and CpGs loading and release

**Fig. 3:**
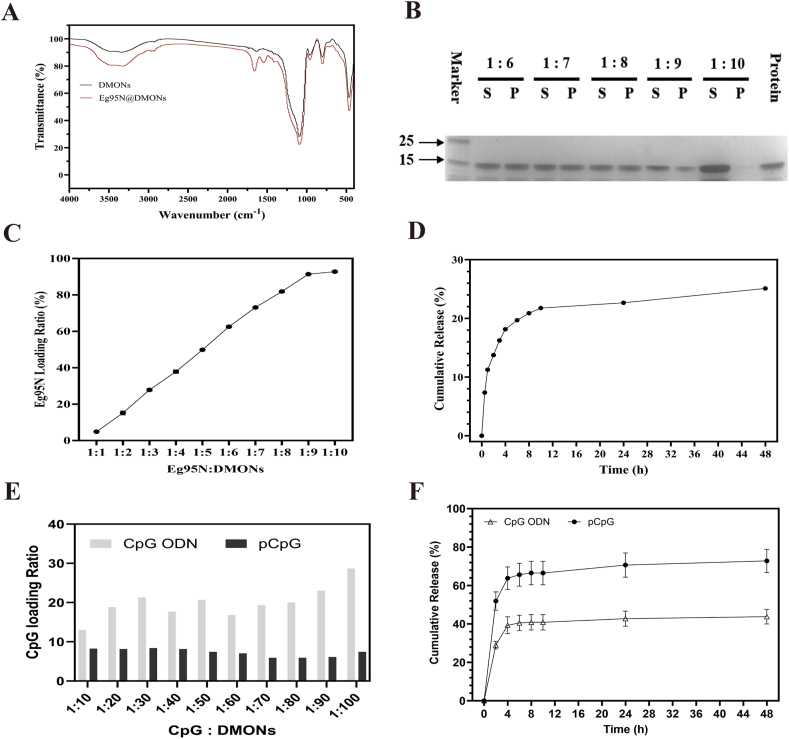
The loading and release characteristics of Eg95N antigen. (A) FTIR spectra of DMONs and Eg95N@DMONs. (B) SDS-PAGE analysis. S: soluble Eg95N in supernatant; P: Eg95N@DMONs in precipitation. (C) The loading ratio of Eg95N at varying mass ratios. A constant of concentration of 250 μg/mL Eg95N and the DMONs with gradient concentration spanning 250–2500 μg/mL were employed in this test. (D) The cumulative release curve of Eg95N from Eg95N@DMONs in PBS solution at 4 °C for 48 h. (E) The loading ratio of CpGs at varying mass ratios. A constant of concentration of 100 μg/mL CpG ODN or pCpG and the DMONs with gradient concentration spanning 1000–10000 μg/mL were employed. (F) The cumulative release curve of CpGs from CpGs @DMONs in PBS solution at 4 °C for 48 h.

The loading capacity of the nanomaterials is an important indicator to evaluate their use as vaccine delivery systems. In this study, the loading and release characteristics of Eg95N protein from Eg95N@DMONs were assessed, and the results were shown in Fig. 3. First, The FTIR spectra of Eg95N@DMONs and DMONs were characterized. The results showed that the characteristic peak at 1080 cm^−1^ remained prominent after loading, signifying that the silicon material continued to play a dominant role in the composite structure. Meanwhile, newly emerged absorption peaks at 2970 cm^−1^, 2920 cm^−1^, 1660 cm^−1^, and 1540 cm^−1^ were observed post-loading. These peaks correspond to the vibrational absorption of carboxyl and amide groups within the Eg95 protein, thereby providing compelling evidence that the Eg95 protein had been successfully adsorbed onto the DMON material (Fig. 3A). Second, the loading capacity of DMONs for the Eg95 protein was evaluated by BCA method. DMONs and Eg95 protein, both at concentrations of 1 mg/mL, were mixed in a 1:1 volumetric ratio and incubated at 4 °C for 14 h. After centrifugation, the protein concentration in the supernatant was measured as 266 μg using Pierce BCA Assay Kit. The loading capacity of DMONs for the Eg95 protein was calculated to be as high as 468 μg/mg. This remarkable loading efficiency can be attributed to its structural characteristics of large surface area and high pore volume, which surpassing previously reported MSNs-based nanocarriers. The optimal combination ratio between Eg95N and DMONs was determined and the results showed that the soluble Eg95N in the supernatant gradually increased as the Eg95N: DMONs ratio progressively decreased (Fig. 3B). The loading ratio of Eg95N exceeded 92 % (Fig. 3C) at the ratio of 1:10, which was subsequently employed as the standard parameter for vaccine formulation. The combination ratio between CpGs and DMONs was also measured. The results showed that the loading ratios of CpG ODN and pCpG were both lower than that of Eg95N at different ratios, not exceeding 30 % and 10 %, respectively (Fig. 3E). When the vaccine formulation was used for co-loading, the loading efficiency of CpGs was significantly improved (Supplementary Fig. 1). We speculate that this is due to the interaction between the positively charged Eg95N (pI = 9.3) and the negatively charged CpGs. Finally, the release profile of Eg95N from Eg95N@DMONs was characterized by an initial rapid release (20 % within 6 h), followed with only 5 % cumulative release over the subsequent 42 h (Fig. 3D). For CpGs, the release profiles of CpG ODN and pCpG from CpGs@DMONs were characterized by a rapid release within 6 h (40 % for CpG ODN; 65 % for pCpG), followed with only 2 % (CpG ODN) and 5 % (pCpG) cumulative release over the subsequent 42 h (Fig. 3F). This favorable sustained release profile of DMON to Eg95N and CpGs may contribute to the antigen/adjuvant depot effect and thereby potentially confer a long-term immunity *in vivo*.

### 3.4 DMONs-mediated antigen and CpGs delivery

**Fig. 4:**
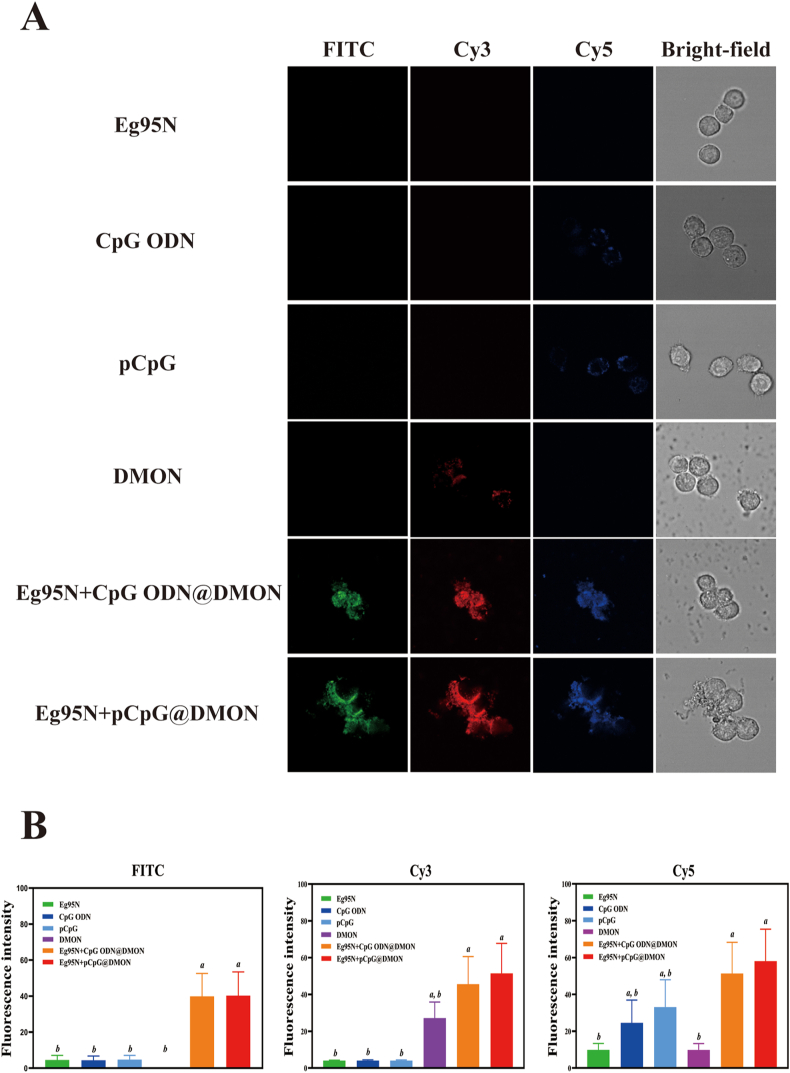
The DMON facilitated the cellular uptake of the Eg95N and CpGs. (A) Confocal microscopy images of RAW264.7 cells incubated with Eg95N-FITC, CpG ODN-Cy5, pCpG-Cy5, DMON-Cy3, Eg95N-FITC + CpG ODN-Cy5@DMON-Cy3, or EG95N-FITC + pCpG-Cy5@DMON-Cy3 at 37 °C for 3 h. (B) Fluorescence intensity of FITC, Cy3 and Cy5 were quantified. Data are presented as the Mean ± SD. One-way ANOVA followed by Turkey multiple comparison was used for statistical analysis, different letters indicate statistically significant differences (*p* < 0.05).

The cellular uptake of soluble Eg95, CpGs, and their DMON-loaded forms were assessed and compared by laser scanning confocal microscopy. After treatment with Eg95N-FITC, CpG ODN-Cy5, pCpG-Cy5, DMON-Cy3, Eg95N-FITC + CpG ODN-Cy5@DMON-Cy3, or EG95N-FITC + pCpG-Cy5@DMON-Cy3, RAW264.7 cells showed viable state with normal morphology in brightfield image. The intracellular red and blue fluorescent dots (corresponding to DMON and CpGs, respectively) were clearly observed, whereas no green fluorescence signal representing Eg95N could be detected, indicating soluble CpGs were able to be taken up by immune cells more easily or faster than Eg95 protein. When antigen and adjuvant(s) were co-loaded onto DMON, their corresponding fluorescence signals were all enhanced visually (Fig. 4A, Supplementary Fig. 2). Quantitative analysis revealed that the FITC signal of Eg95N-FITC + CpG, ODN-Cy5@DMON-Cy3 and EG95N-FITC + pCpG-Cy5@DMON-Cy3 was significantly higher than that of soluble Eg95N-FITC (Fig. 4B), while no significant difference of Cy5 fluorescence intensity between CpGs-Cy5 and Eg95N-FITC + CpGs-Cy5@DMON-Cy3 was observed (Fig. 4B), indicating DMON exerts a stronger mediating effect on protein internalization than on CpGs. These findings supported DMON has the property of being easily taken up by immune cells, which can be used for efficient delivery of its loaded Eg95N antigen and CpG adjuvant(s) into immune cells.

**Fig. 5:**
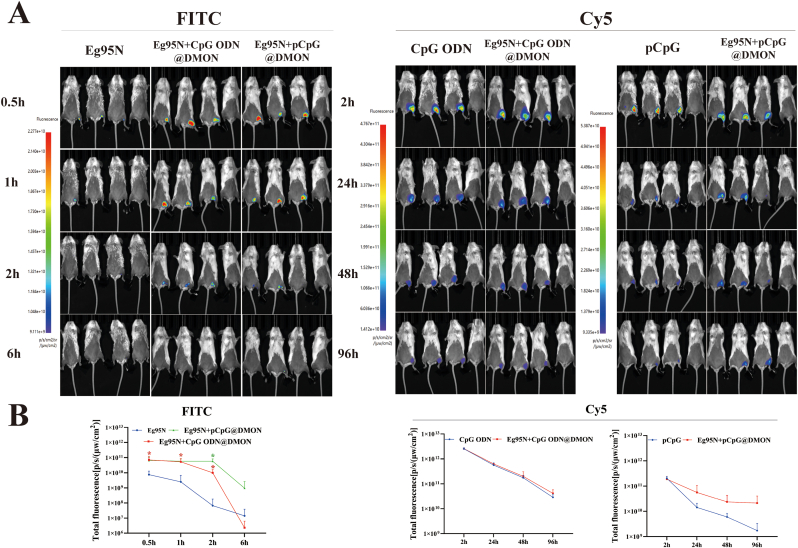
The DMON facilitated the deposit or delivery of Eg95N and CpGs to lymph nodes. (A) Mice were immunized intramuscularly in thigh with 0.1 mL of Eg95N-FITC (30 μg), pCpG-Cy5 (10 μg), CpG ODN-Cy5 (10 μg), Eg95N-FITC + pCpG-Cy5@DMON or Eg95N-FITC + CpG ODN-Cy5@DMON and then imaged at 0.5 h (2 h), 1 h (24 h), 2 h (48 h), and 6 h (96 h) using IVIS. The fourth mouse (rightmost) in each photo of the figure served as a control to adjust the fluorescence intensity range. (B) Fluorescence intensity of FITC and Cy5 were quantified. Data are presented as the Mean ± SD. Unpaired two-tailed *t*-test was used for statistical analysis, *p* < 0.05 indicates statistically significant differences.

Next, whether the DMON could facilitate antigen/adjuvant retention or delivery was examined by IVIS Spectrum. The DMON-mediated *in vivo* trafficking kinetics of Eg95N and CpGs was shown in Fig. 5. Overall, the intensity and retention time of FITC signals were lower than those of Cy5, which may be determined by the characteristics of FITC that its relatively short emission wavelength leads to poor penetrability *in vivo* imaging. For Eg95N antigen, the FITC fluorescence signals of the two vaccine groups were obviously stronger than those of Eg95N group at the injection site (Fig. 5A), accompanied by significant differences at certain time points (0.5 h, 1 h, and 2 h) (Fig. 5B); meanwhile, fluorescence signals were observed near the inguinal lymph nodes in the vaccine groups (Eg95N + pCpGs@DMON and Eg95N + CpG ODN@DMON). For CpGs, the Cy5 fluorescence signals of the vaccine groups were higher than those of the CpGs groups (24 h, 48 h, 96 h) (Fig. 5A), but there was no significant difference (Fig. 5B); meanwhile, a trend of the migration of the Cy5 fluorescence signals towards the lymph nodes was also observed in two vaccine groups, especially in Eg95N + pCpGs@DMON. These findings demonstrated that DMON not only facilitates the antigen depot, but also accelerates the delivery of its cargos to the lymph nodes, aiding the antigen-processing and immune activation. Taken together, the DMON can serve as an effective co-delivery system for Eg95 and CpGs, highlighting its potential in the design of our ‘all-in-one’ vaccine that probably confers the potent and durable immune response we expect.

### 3.5 Efficacy evaluation of DMONs-scaffolded vaccines

#### 3.5.1 Humoral immune responses of the DMONs-scaffolded vaccines

**Fig. 6:**
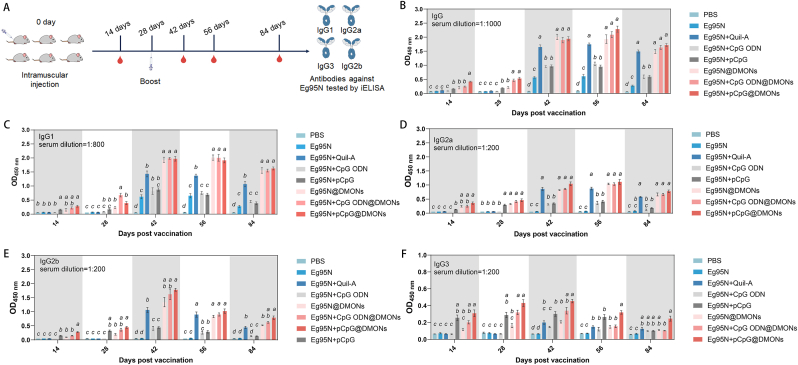
Humoral immune responses induced by different vaccines. (A) Schematic of the immune procedure. Mice were vaccinated on day 0 at the right femoral muscle and boosted on day 28 at the left femoral muscle. Serum sample were collected at 14, 28, 42, 56, and 84 dpv. Levels of Eg95-specific IgG (B), IgG1 (C), IgG2a (D), IgG2b (E), IgG3 (F) were measured by indirect ELISA. Data are presented as the Mean ± SEM. One-way ANOVA followed by Turkey multiple comparison was used for statistical analysis, different letters indicate statistically significant differences (*p* < 0.05).

Healthy BALB/c mice were intramuscularly injected with vaccines from seven groups (Table 1) or negative control and the serum samples were harvested according to the immunization schedule (Fig. 6A) for detecting Eg95-specific antibodies. Overall, PBS (negative control) failed to induce Eg95-specific antibody responses throughout the entire experimental period, demonstrating the high specificity of the method (Fig. 6B–F).

For total serum IgG, the Eg95-specific IgG responses across groups followed a similar trend, peaking at 56 dpv. Specifically, at 42, 56 and 84 dpv, the IgG responses induced by Eg95N + Quil-A, Eg95N + CpG ODN, Eg95N + pCpG, and Eg95N@DMONs were significantly higher than that of the sole Eg95N, and the IgG responses in Eg95N + Quil-A and Eg95N@DMONs were also significantly higher than those in the other two groups, indicating strong adjuvanticity to the Eg95N antigen of these adjuvants (including three potentiators: Quil-A, pCpG, CpG ODN and one nanocarrier: DMON), with DMON showing the best performance. Strikingly, Eg95N + pCpG and Eg95N@DMONs triggered significantly higher responses than Eg95N + Quil-A and Eg95N + CpG ODN at 14, 28 dpv, suggesting the more potent early-stage adjuvanticity of pCpG and DMON. When combining these two types of adjuvants (CpG ODN/pCpG + DMON), the best profile with anticipatory, enhanced and endurable IgG responses compared to other vaccines were observed in Eg95N + CpGs@DMONs, with Eg95N + pCpG@DMONs outperforming Eg95N + CpG ODN@DMONs (*p* < 0.05, at 14 dpv).

To comprehensively understand the antibody response profile, four IgG isotypes were also analyzed (Fig. 6C–F). Comparing the OD value and dilution ratio, IgG1 constituted the highest proportion of total IgG (60 %–65 %), while IgG3 response was the weakest among all detected IgG isotypes. Overall, IgG isotypes responses exhibited similar kinetics to the total IgG response, with notable differences observed as follows. First, DMONs-scaffolded nanovaccines including Eg95N@DMONs, Eg95N + CpG ODN@DMONs, and Eg95N + pCpG@DMONs induced significantly higher IgG1 response than the positive control (Eg95N + Quil-A) at 42, 56, and 84 dpv. Second, Eg95N + pCpG@DMONs elicited significantly higher IgG2b responses at 42, 84 dpv and obviously stronger IgG3 responses at 42, 56 dpv, compared to the positive control (Eg95N + Quil-A). These isotype-specific variations reflected the functional diversity among IgG isotypes, yet did not alter the overarching conclusion that Eg95N + pCpG@DMONs elicited the most potent humoral immunity.

#### 3.5.2 Cellular immune responses of the DMONs-scaffolded vaccines

**Fig. 7:**
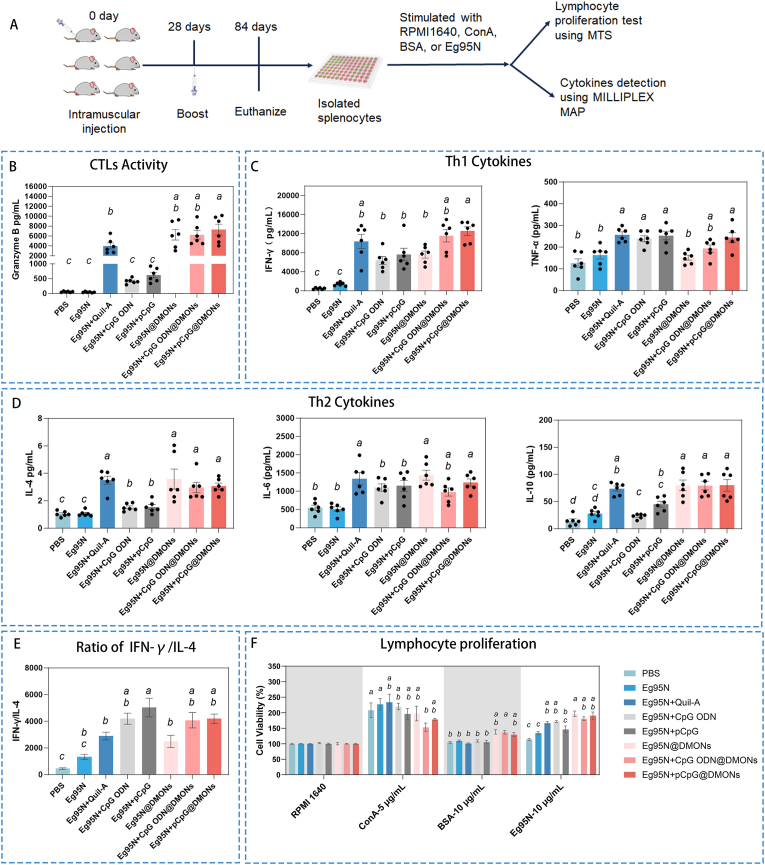
Cellular immune responses induced by different vaccines. (A) The flow chat of the preparation process of lymphocyte proliferation and Granzyme B/cytokines determination. The secretion levels of Granzyme B (B), Th1 (C) and Th2 (D) cytokines. (E) The ratio of signature Th1 and Th2 cytokines. (F) Lymphocyte proliferation analysis. Data are presented as Mean ± SEM. One-way ANOVA followed by Turkey multiple comparison was used for statistical analysis, different letters indicate statistically significant differences (*p* < 0.05).

The levels of Granzyme B and cytokines secreted by splenocytes stimulated with Eg95N antigen for 72 h were quantified (Fig. 7A). The highest level of Granzyme B was observed in DMONs-scaffolded vaccines (Eg95N@DMONs, Eg95N + ODN@DMONs, and Eg95N + pCpG@DMONs), among them, Eg95N + pCpG@DMONs induced significantly higher (*p* < 0.05) Granzyme B production than positive control (Eg95N + Quil-A) (Fig. 7B). These results suggested that the adjuvant combination (pCpG + DMON) was able to synergistically enhance Eg95-induced cytotoxic T lymphocyte (CTL) activation.

For Th1 cytokines (Fig. 7C), the secretion levels of IFN-γ in Eg95N + pCpG@DMONs, Eg95N + CpG ODN@DMONs, as well as positive control (Eg95N + Quil-A) were markedly elevated compared to other groups; the secretion levels of TNF-α were generally low in all groups, and all three potentiators (pCpG, CpG ODN, Quil-A) could enhance the TNF-α secretion (Eg95N + pCpG/Eg95N + CpG ODN/Eg95N + Quil-A *vs* Eg95N), while nanocarrier DMONs could not (Eg95N@DMONs *vs* Eg95N). For Th2 cytokines (Fig. 7D), the IL-4, IL-6 and IL-10 levels in DMONs-scaffolded vaccines (Eg95N@DMONs, Eg95N + CpG ODN@DMONs, Eg95N + pCpG@DMONs) were comparable to those in positive control Eg95N + Quil-A (*p* > 0.05). The ratio of IFN-γ/IL-4 was calculated and no significant difference between DMONs-scaffolded vaccines and Eg95N + Quil-A was observed (*p* > 0.05) (Fig. 7E).

Antigen-specific T cell activation and proliferation were measured via MTS assay. As shown in Fig. 7F, the proliferation response to Eg95N in spleen lymphocytes from mice immunized with Eg95N + Quil-A, Eg95N + CpG ODN, Eg95N@DMONs, Eg95N + CpG ODN@DMONs, or Eg95N + pCpG@DMONs, exhibited similar proliferation responses to Eg95 stimulation, all of which were significantly higher than that in the sole Eg95N group, indicating the proliferation of Eg95 auto-reactive T cells, could be activated obviously by all adjuvants except pCpG *in vivo*.

Taken together, when potentiator CpGs and delivery system DMON were co-employed as a combination adjuvant, the advantages of both types of adjuvants were synergized and complemented. The designed ‘all in one’ DMON-scaffolded vaccines (Eg95N + CpGs@DMONs) were capable of inducing significant humoral and cellular immune responses, which may confer early, robust and durable efficacy against CE, given that and the level of Eg95-specific antibodies are directly related to the protective immunity [29,[32], [33], [34], [35]]. However, the challenge experiments in mouse model or even in sheep, which can directly evaluate the protective efficacy of the novel vaccine, need to be addressed in the future, when the authorized biosafety level-3 laboratory is available for us.

### 3.6 Safety evaluation

#### 3.6.1 Safety evaluation *in vitro*

**Fig. 8:**
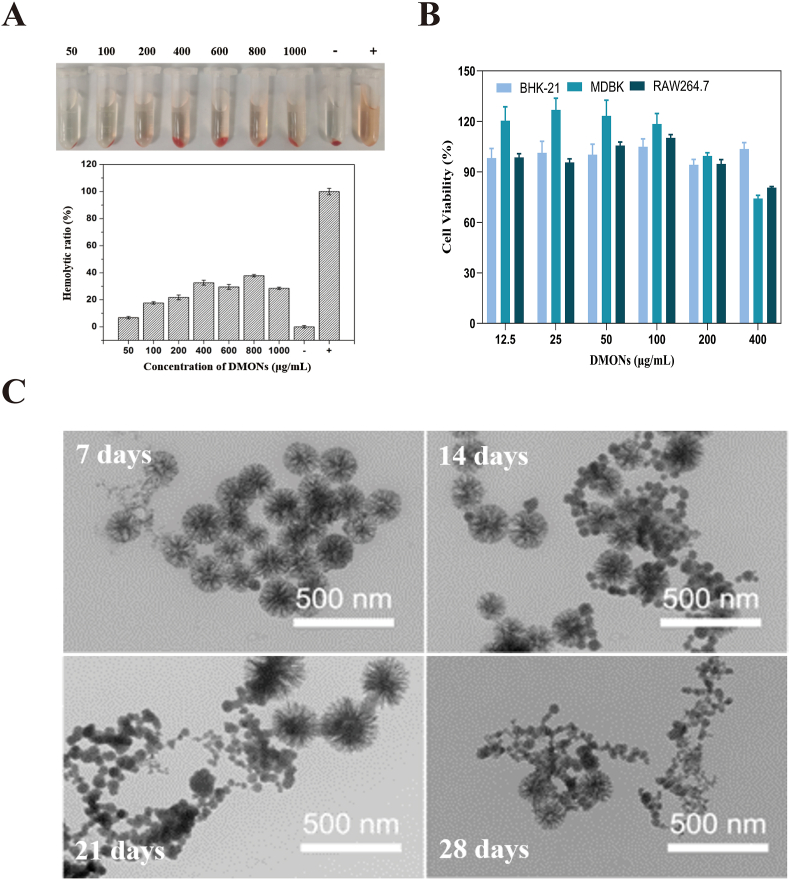
Safety evaluation of DMONs *in vitro*. (A) Hemolysis assay of DMONs with different concentrations, and UV–vis absorption spectroscopy of the hemoglobin released from RBCs of guinea pig. (−) and (+) controls represent hemolytic ratios of the RBCs in PBS and in water, respectively. (B) Cell viability of BHK-21, MDBK, and RAW 264.7 cell lines following incubation with DMONs at gradient concentrations was determined using the MTS assay. The data are presented as the Mean ± SEM. One-way ANOVA followed by Turkey multiple comparison was used for statistical analysis. (C) Time-dependent TEM imaging of DMONs in SBF solution.

The possible side effects of administering the DMON-scaffolded vaccines were evaluated *in vitro* and *in vivo.* The hemolytic effect, associated with biocompatibility, was evaluated using RBCs exposed to DMONs with gradient concentrations (50, 100, 200, 400, 800, and 1000 μg/mL). The results showed that the hemolytic activity of DMON does not increase significantly with increasing concentration, and remained below 40 % even at 800 μg/mL (Fig. 8A), indicating good blood compatibility of DMONs. The cytotoxicity of the DMONs was evaluated in BHK-21, MDBK, and RAW264.7 cell lines by MTS assay (Fig. 8B). The results showed that BHK-21, MDBK, and RAW264.7 cells showed over 94 % viability at a high dose of DMONs (200 μg/mL), and still maintained over 70 % (103.56 %, 74.17 %, 80.67 %, respectively) even at the highest dose (400 μg/mL), indicating negligible cellular toxicity of DMONs. The biodegradability of the DMONs was evaluated in simulated body fluid (SBF). After 28-day incubation at 37 °C, DMONs exhibited near-complete structural collapse, with concomitant size reduction (Fig. 8C). These findings demonstrate excellent biodegradability and low *in vivo* accumulation potential of DMONs.

#### 3.6.2 Safety evaluation *in vivo*

**Fig. 9:**
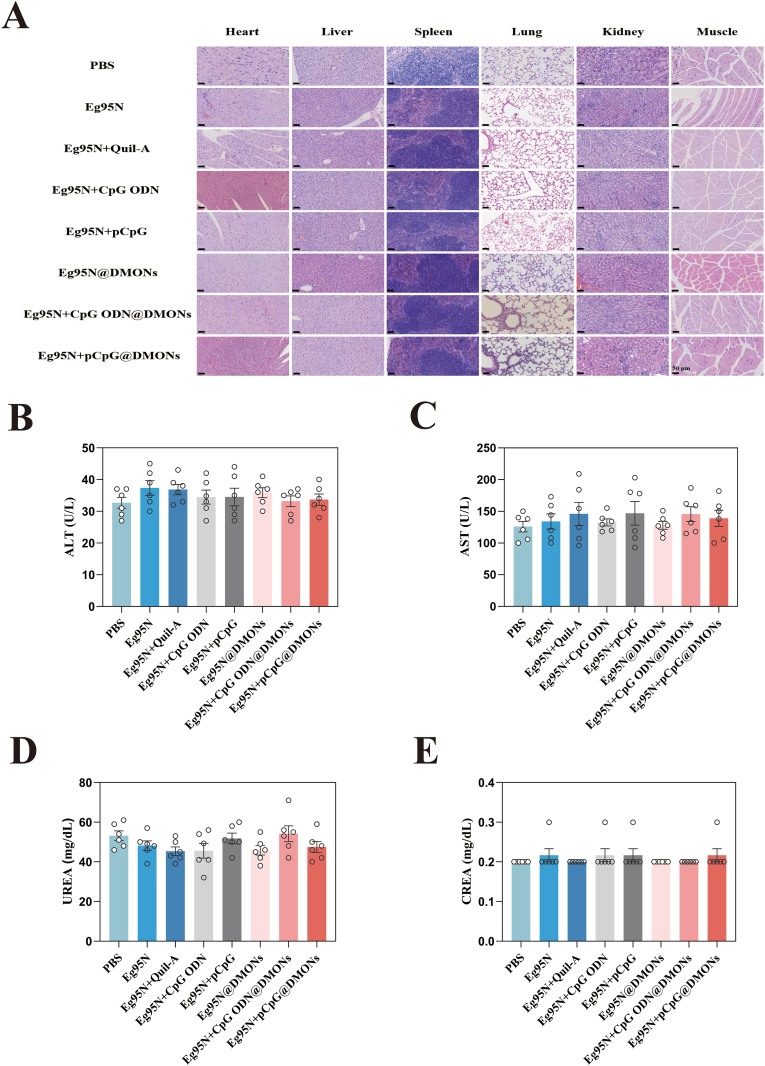
Safety evaluation of vaccines *in vivo*. (A) H&E staining images of different organs harvested from vaccinated mice at 84 dpv. Scale bars, 50 μm. The concentrations of ALT (B), AST (C), UREA (D) and CREA (E) were measured by biochemical test using serum collected at 84 dpv. The data are presented as the Mean ± SEM. One-way ANOVA followed by Turkey multiple comparison was used for statistical analysis.

Histopathological analysis revealed no significant pathological abnormalities in all tissue samples from mice administered with Eg95N + CpGs@DMONs nanovaccines compared to controls (Fig. 9A). Serum biochemical indicators were also monitored after intramuscular inoculation, and showed comparable levels of alanine aminotransferase (ALT), aspartate aminotransferase (AST), urinary albumin excretion rate (UREA) and creatinine (CREA), suggesting the absence of obvious hepatotoxic or nephrotoxic effects induced by DMONSs-based vaccines (Fig. 9B–E).

Collectively (section 3.7), *in vivo* and *in vitro* safety evaluations demonstrated negligible side effects, confirming the excellent safety profiles of DMON.

## 4 Conclusion

The DMON with a distinctive center-radial were successfully developed, and employed as an efficient nanocarrier for Eg95 antigen and CpG adjuvants delivery, resulting in the novel vaccine Eg95N + CpGs@DMONs against echinococcosis. This DMON-scaffolded nanovaccine, with sustained release, cellular uptake and *in vivo* transport capacities for its cargos, exhibited excellent safety profiles, and elicited an early-onset, potent and long-lasting protective immunity compared to the commercial one. The combination adjuvant (DMON + CpGs) system-based nanovaccines present good potential to upgrade current livestock-used vaccine and show favorable applicability for humans.

## CRediT authorship contribution statement

**Ting Xin:** Writing – review & editing, Writing – original draft, Visualization, Software, Methodology, Investigation, Data curation. **Xintao Gao:** Writing – review & editing, Writing – original draft, Visualization, Software, Methodology, Investigation, Data curation. **Siyi Tao:** Software, Methodology, Data curation. **Chenghao Zhou:** Visualization, Software. **Zhifang Zhang:** Validation, Supervision, Funding acquisition, Data curation. **Jiabo Ding:** Validation, Supervision, Data curation. **Jiaxi Ru:** Writing – review & editing, Writing – original draft, Visualization, Validation, Supervision, Resources, Project administration, Conceptualization. **Yinü Li:** Writing – review & editing, Validation, Supervision, Resources, Project administration, Funding acquisition, Conceptualization.

## Declaration of competing interest

The authors declare that they have no known competing financial interests or personal relationships that could have appeared to influence the work reported in this paper.

## Data Availability

Data will be made available on request.
