# Supplementary material for: A tailored nanocarrier DMON and CpGs synergistically drive the formulation of a highly immunogenic and long-acting vaccine against echinococcosis
Source: Mater Today Bio. 2025 May 12;32:101868. doi: 10.1016/j.mtbio.2025.101868 (PMC12144504; doi:10.1016/j.mtbio.2025.101868)
Supplement: Multimedia component 1 [file mmc1.docx]

**Supplementary materials**


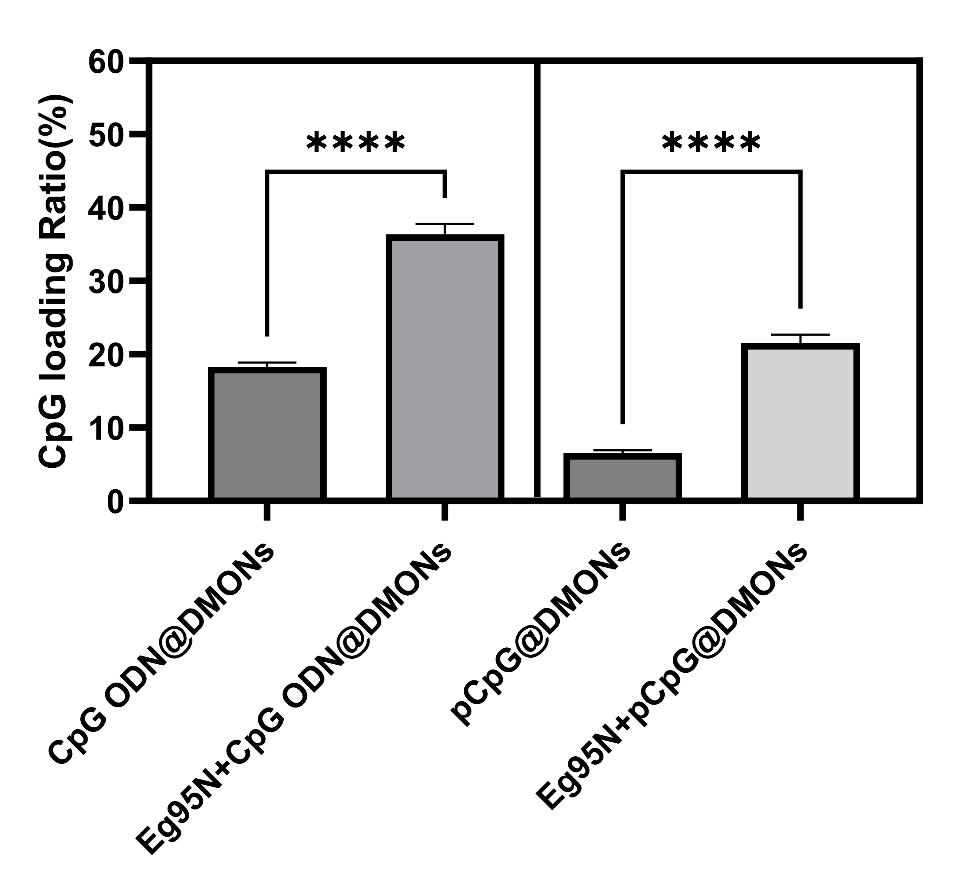


Supplementary Figure 1. Eg95N promotes the loading ratio of CpGs onto DMON. Vaccine formulations (Eg95N: 0.15 mL, 1 mg/mL; CpG ODN: 0.05 mL, 1 mg/mL; pCpG: 0.05 mL, 2 mg/mL) were employed. Data are presented as the Mean ± Sem. One-way ANOVA followed by Turkey multiple comparison was used for statistical analysis, different letters indicate statistically significant differences (*p* < 0.05).


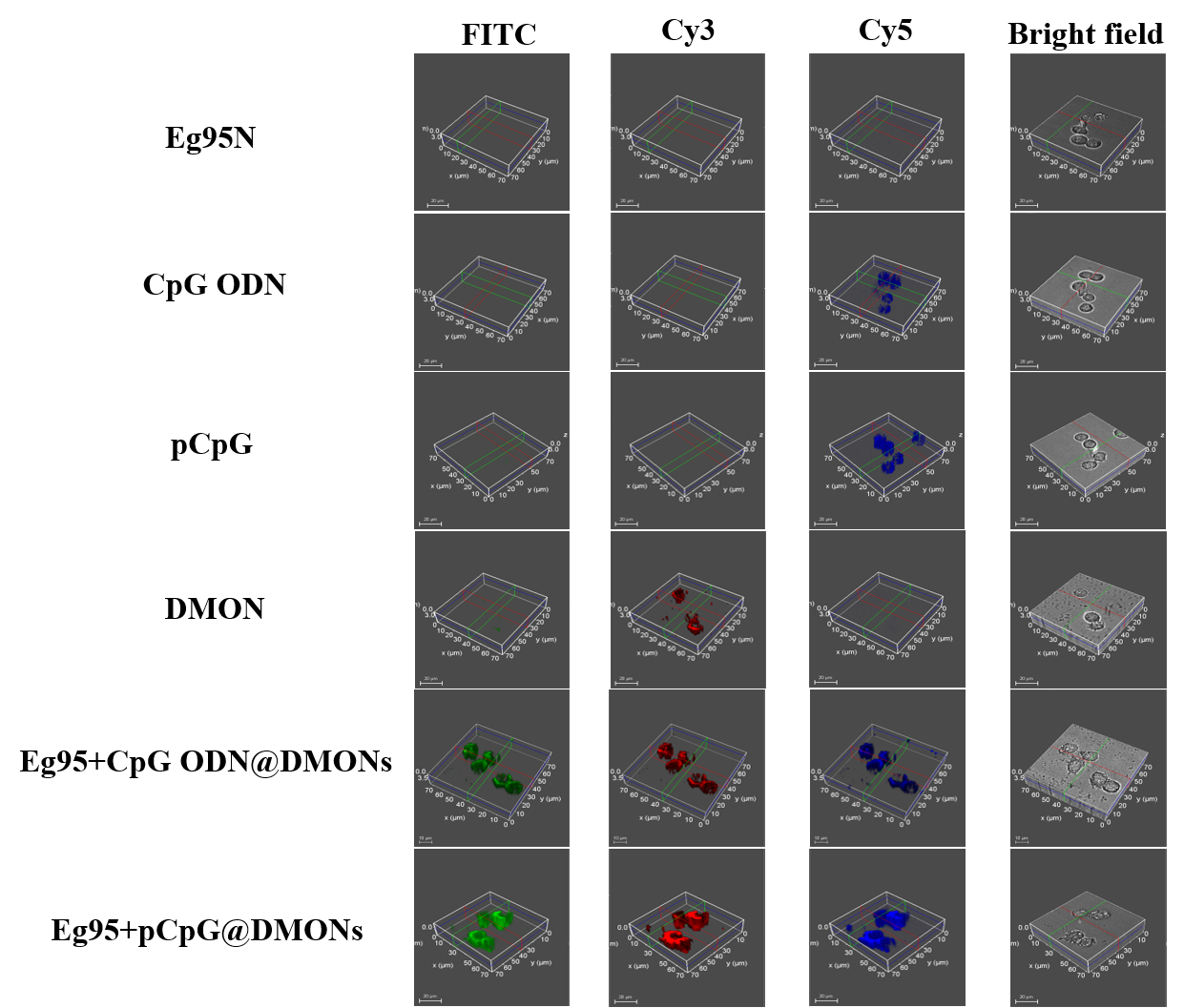


Supplementary Figure 2. 3D reconstruction fluorescence images of RAW264.7 cells incubated with Eg95N-FITC, CpG ODN-Cy5, pCpG-Cy5, DMON-Cy3, Eg95N-FITC+CpG ODN-Cy5@DMON-Cy3, or EG95N-FITC+pCpG-Cy5@DMON-Cy3 for 3 h.
